# Groundwater Potential Zone Mapping Using Analytical Hierarchy Process and GIS in Muga Watershed, Abay Basin, Ethiopia

**DOI:** 10.1002/gch2.202100068

**Published:** 2021-10-15

**Authors:** Tadele Melese, Tatek Belay

**Affiliations:** ^1^ Department of Natural Resource Management College of Agriculture and Environmental Sciences Bahir Dar University Bahir Dar 5501 Ethiopia; ^2^ Department of Geography and Environmental Studies College of Social Science Debre Tabor University DebreTabor 273 Ethiopia

**Keywords:** GIS, groundwater prospect, Muga watershed, receiver operating characteristic curve (ROC)

## Abstract

Groundwater is an important resource that contributes significantly to the total annual water supply. The purpose of the present study is to assess and delineate the groundwater recharge zone using geospatial technology through an analytical hierarchal process (AHP) method in to the Muga watershed, Abay Basin. Remote sensing satellite images and the corresponding data are used for the preparation of thematic layers, viz., geology, rainfall, slope, soil, curvature, topography wetness index, elevation, drainage density, land use land cover, and lineament density of the study watershed. All thematic layers are integrated with a multicriteria evaluation technique. Weighted overlay index analysis is carried out to give rank for each parameter. The weight is assigned for each thematic layer depending on the AHP technique. The reliability of the output is checked by the calculated consistency index and consistency ratio which is reasonably acceptable (0.044 < 0.1). Verification is done by considering the groundwater well locations in the validation datasets. The receiver operating characteristic curve and area under curve (=82.9%) are used to explore the prediction accuracy.

## Graphical abstract

Deciphering groundwater potential zones using a Geographic Information System with analytic hierarchy process method is important to enable administration, management, and sustainable use of groundwater resources in the Muga watershed.

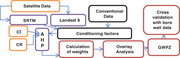

## 1 Introduction

It is undeniable that water has been the most essential natural resource for the substance of life on planet earth. Groundwater is one of the most treasured natural resources, enormously vital and dependable sources of water supply in all climatic regions across the world.^[^ ^1^, ^2^, ^3^, ^4^ ^]^ Groundwater is an important resource contributing significantly to total annual supply.^[^ ^5^, ^6^ ^]^ Uncontaminated and groundwater is a crucial to sustain life on earth, particularly at the present time when water scarcity is becoming central issue in many nations across the globe.^[^ ^7^ ^]^ The tropical and sub‐tropical regions are severely affected with the problems related to groundwater.^[^ ^8^ ^]^

The groundwater resources potential of Ethiopia is estimated to be about 40 billion cubic meters.^[^ ^9^ ^]^ According to ref,^[^ ^10^ ^]^ groundwater provides more than 90% of the water for domestic and industrial supply in Ethiopia, but a very small percentage of water is used for irrigation, which mostly generate from surface water. However, Ethiopia has also suffered frequent devastating droughts with severe ramifications comprising famine, augmented poverty, and civil unrest.^[^ ^10^ ^]^ Overexploitation has exhausted groundwater availability noticeably and also led to land subsidence at some places.^[^ ^11^ ^]^ To control land degradation, long‐term sustainable utilization of water resources, new methods for the controlling of land and water resources are increasing in the present day. Hence, assessing and constructing groundwater potential mapping is very much indispensable for better improvement of groundwater resources and techniques for its investigation.^[^ ^12^, ^13^, ^14^, ^15^, ^16^, ^17^, ^18^, ^19^, ^20^, ^21^, ^22^ ^]^

In the Abay basin, groundwater is a source of domestic water in urban and rural areas.^[^ ^23^ ^]^ According to ref ^[^ ^23^ ^]^ inadequate public water supply is being a serious problem for the community in Muga watershed due to population growth. The practical development of the groundwater resources will have a significant effect on the improvement of the community's livelihood. Generating a groundwater potential map has a significant effect to enhance the sustainable management of groundwater resources in the study area. Thus, the present study is critical for quick identification of groundwater potential for better utility in the study watershed.

Geographic Information System (GIS) is a computer based system that can be used capture, store, manipulate, analyze and present geospatial data to solve several complex and complicated problem in the environment.^[^ ^24^ ^]^ Recently, the application of GIS is being powerful and applied to deciphering the potential areas of groundwater occurrence with cost effective manner. GIS has great role for effectively addressing groundwater exploration and delineating potential areas in a certain region of study. Extensive use of remote sensing satellite images along with ground truth data has made it easier to provide the baseline information for delineating groundwater potential zones. Literatures reveal that several researchers have been using GIS to delineate groundwater potential zones with the integration of statistical approach such as simple additive weight (SAW) and analytic hierarchy process (AHP)^[^ ^25^, ^26^, ^27^, ^28^, ^29^, ^30^, ^31^ ^]^ and, machine learning.^[^ ^32^, ^33^, ^34^, ^35^, ^36^, ^37^, ^38^ ^]^ The combination of GIS and remote sensing technologies reduce the ambiguity of hydrogeological data various aspect.

Recently, several studies have been applied using index‐based models and quantitative approaches for assessing groundwater potential zones.^[^ ^39^, ^40^, ^41^, ^42^, ^43^, ^44^, ^45^, ^46^, ^47^, ^48^, ^49^ ^]^ AHP is the most popular and widely used of Multi‐Criteria Decision Analysis (MCDA) techniques to delineate groundwater prospecting zones.^[^ ^42^, ^44^, ^45^ ^]^ Consequently, the AHP technique was used for this study to assign the relative importance of each parameter for zoning groundwater potential recharging area.

A few studies have been conducted in Abay basin to delineate the potential zones of groundwater occurrence.^[^ ^23^, ^47^ ^]^ However, the causative factors such as rainfall, curvature, topography wetness index, and elevation did not consider under the study. Rainfall is one of the main sources and an important factor of groundwater recharge.^[^ ^18^ ^]^ Elevation and TWI have been widely used for groundwater potential exploration and to explain hydrogeologic conditions.^[^ ^45^ ^]^ In the present study, the aforementioned excluded causative factors have been used to decipher groundwater potential zones with integration of AHP method in GIS environment. The main objective of this study was to assess and delineate groundwater recharge zone using Geospatial technology through AHP method. The output of this research will provide valuable information to develop sustainable groundwater management and suitable location for borehole drilling that can be used by decision‐makers, government agencies, and private sectors. Furthermore, the results of this study important to have proper administration, management, and sustainable use of groundwater resources in Muga watershed.

## 2 Experimental Section

### 2.1 Description of the Study Area

**Figure 1:**
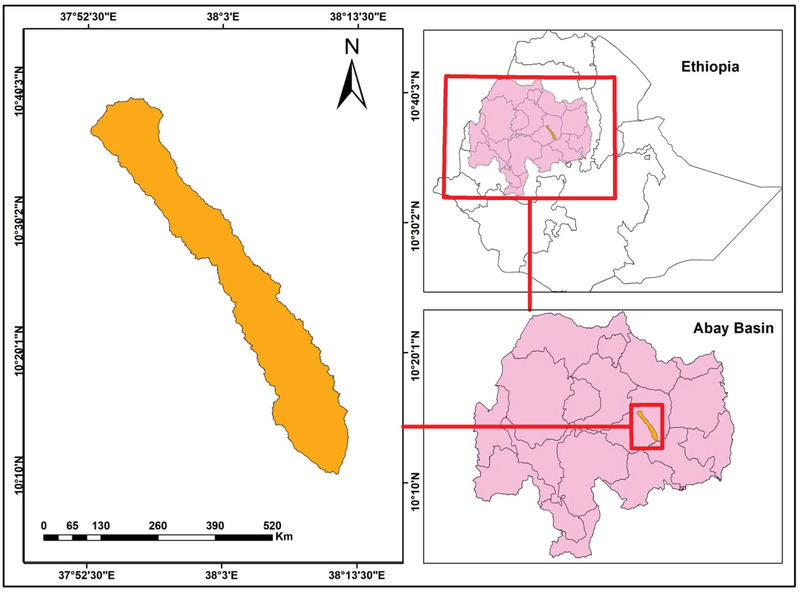
Location map of the study watershed.

The study watershed lies in between 10° 05′ N to 10° 43′48″N and 10° 49′12″ E to 38° 8′56″ E (**Figure** **1**). The total areal extent of the study watershed is 705 km^2^, which is located in the southeastern part of Mount Choke in the headwater area of Abay basin in Ethiopia.^[^ ^49^, ^50^ ^]^ The topographical elevation ranges from about 2384–4088 m above mean sea level. Soil groups which are found in the study area are Chromic luvisols, chromic vertisols, eutric cambisols, eutric nitosols, leptosols, and pellic vertisols.^[^ ^23^ ^]^

The dominant land use land cover comprises cultivated, wetland, grassland, shrubland, bare land, and forest The economic activity of the study area dependent on agriculture practices and there is a huge demand of groundwater for irrigation purposes.

## 3 Materials and Methods

**Figure 2:**
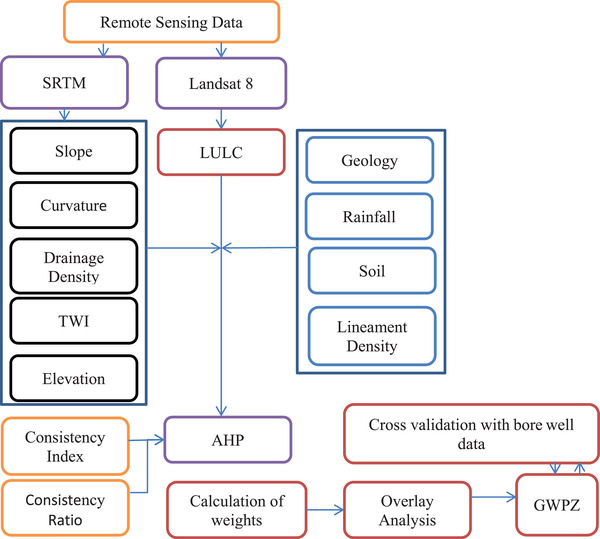
Flow chart of the methodology.

In this study, various types of data were used to delineate groundwater possible areas in the study watershed. A digital elevation model (DEM) with a 30 m resolution was obtained from Shuttle Radar Topography Mission (SRTM) to derive a slope and drainage density map using the ArcGIS tool. Remote sensing satellite images and the corresponding data have been carried out for the preparation of thematic layers viz., geology, rainfall, slope, soil, curvature, topography wetness index, elevation, drainage density, land use land cover, and lineament density of the study watershed. All thematic layers were integrated with Multicriteria evaluation technique. The potential zones of groundwater were obtained by overlaying all thematic layers based on weighted overlay method. Weighted overlay index analysis was carried out to give rank for each parameter of each thematic layer. The weight was given for each thematic layer depending on the Analytic hierarchy process (AHP) technique. These thematic layers were then subjected to a weighted overlay analysis and the final resulting map is acquired and classified based on the groundwater potential index determined (**Figure** **2**).

### 3.1 Data Development for Parameters Related to Groundwater Potential

Several thematic layers that favor the groundwater occurrence have been combined and a groundwater potential map has been prepared in GIS. The thematic maps (Factor maps) used for the groundwater prospective mapping in this study are briefly described below.

#### 3.1.1 Drainage Density

**Figure 3:**
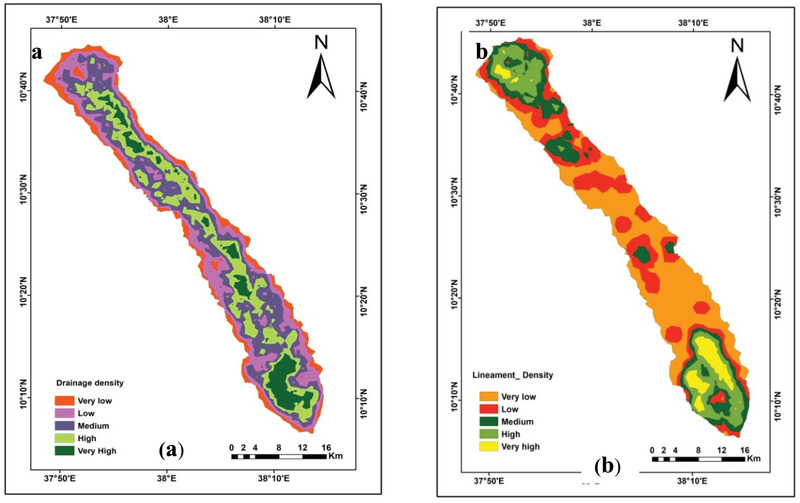
a) Drainage density and b) lineament density of the study area.

Drainage density indicates the closeness of spacing of stream channels and can be calculated as the total length of all the streams and rivers in the watershed divided by the total area of drainage watershed. The drainage density has an inverse relationship with groundwater prospect. The higher the drainage density is the lower the probability of groundwater potential zone.^[^ ^51^ ^]^ Hence, the kernel density method in Arc GIS has been carried out to calculate drainage density. The drainage network within the area was extracted from the DEM and updated from the satellite image (**Figure** **3**a). Hence, the kernel density method in Arc GIS has been carried out to calculate drainage density using formulae as given below^[^ ^52^ ^]^ The map shows that most of the study area is covered by moderate to low drainage density that refers to more infiltration and recharge to the groundwater

(1) $$\begin{array}{c} \text{DD}=\sum \frac{\text{LWS}}{\text{AWS}} \end{array}$$

where DD is the drainage density, LWS is the total length of streams in the watershed, and AWS is the area of a watershed.

#### 3.1.2 Lineament Density

A lineament is a linear feature in a geographical landscape that is a manifestation of fundamental geological structure.^[^ ^53^, ^54^ ^]^ The lineament density was defined as the total length of all the recorded lineaments divided by the area under consideration.^[^ ^55^ ^]^ The lineament density map of Muga watershed was prepared by using the line feature collected from the Geological Survey of Ethiopia. The present study used lineament density, which represents the total length of lineament as a unit area, as expressed^[^ ^23^, ^56^ ^]^

(2) $$\begin{array}{c} \text{Ld}=\frac{\sum_{i=1}^{i=n}Li}{A} \end{array}$$

where $\sum_{i=1}^{i=n}Li$ denotes the total length of lineaments and *A* denotes the unit area [*L*2].

Lineament density is directly proportional to the groundwater recharge zone. The purpose of the lineament analysis is to improve understanding of the relationship between the surface water penetration and fracture systems, controlling water infiltration and mobility. The lineament density map shows that the upper and lower part of the studied watershed was considered an excellent and promising groundwater zone (Figure 3b).

#### 3.1.3 Slope

The amount of water available for recharge and the ruggedness of the terrain of any watershed is described by the slope of that watershed. A large volume of runoff and lower infiltration are related to regions with steep angles of elevation.^[^ ^57^ ^]^ Hence, slope is one of the influential factors affecting runoff and infiltration rate. The slope of the present study was developed from SRTM DEM. Weights for each slope class were assigned based on the level of groundwater potential.

**Figure 4:**
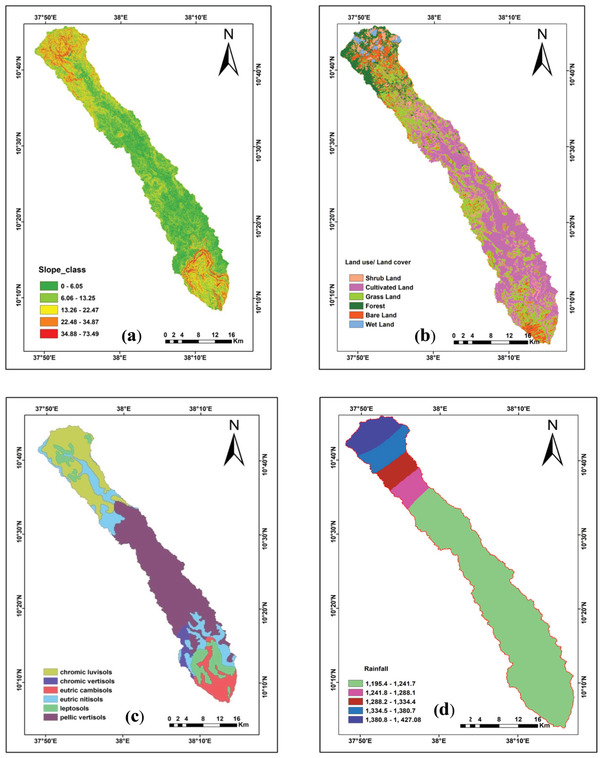
a) Slope class, b) land use land cover types, c) soil types, and d) rainfall map of Muga watershed.

For the effect of assigning ranks, the slope of the region is categorized into five categories. The highest rating was assigned to the flat terrain with a slope value 0–6.05, and the ratings were gradually decreased as the slope value increased. The steeper slope value ranging from 34.88–73.49 with the lowest rating of 0.03 was found in the upper and lower escarpment part of the study watershed as depicted in **Figure** **4**a.

#### 3.1.4 Land Use Land Cover

Land use land cover (LULC) is an important factor affecting groundwater recharge, groundwater occurrence, and availability.^[^ ^58^ ^]^ LULC map data was derived from the Landsat 8 (OLI) satellite image of 2021 with 30‐ m spatial resolution. Supervised image classification was conducted to classify and identify the type of LULC.

The study area consists of seven types of LULC (Figure 4b) namely; cultivated land, shrubland, grassland, forest, bare land, and wetland. One of the dominant land use/land cover categories in the study area is cultivated land followed by grassland. Cultivated land (irrigated cropping) is considered to be the most suitable zone for recharge as it favors the percolation of rainwater as well as irrigated water. The recharging rate of groundwater in irrigated cropping is substantially increases in all cases, particularly marked where particularly flood irrigation practiced.^[^ ^59^ ^]^

#### 3.1.5 Soil

Soil types of the area are playing a significant role in groundwater recharge and water holding capacity of the area. Consequently, it could be considered as one of the important factors for the delineation of groundwater potential zones.^[^ ^60^, ^61^ ^]^ The major soils found in the study area are Chromic luvisols, chromic vertisols, eutric cambisols, eutric nitosols, leptosols, and pellic vertisols with loam, sandy loam, clay texture. Weights are assigned subjectively to each soil unit after taking into consideration the type of soil and its water holding capacity. Field checks in the identified soil units were conducted and confirmed (Figure 4c).

#### 3.1.6 Rainfall

Rainfall is an important parameter to delineate groundwater potential and major hydrological sources of groundwater storage.^[^ ^62^, ^63^, ^64^ ^]^ About 85% of the rainfall falls during the rainy season (May to October).^[^ ^50^ ^]^ The amount of rainfall is higher in the upper part of the study area and decreased toward the southern part (Figure 4d). The rainfall map for the study watershed was classified into five categories having maximum and minimum rainfall as 1195 and 1427 mm, respectively. The highest rating was assigned to the Northern (upper escarpment of the watershed) areas receiving the highest rainfall, whereas the rainfall magnitude was found decreasing toward the southern escarpment of the watershed and the rating thus assigned also decreased toward the south direction (lower escarpment of the watershed).

#### 3.1.7 Geology

**Figure 5:**
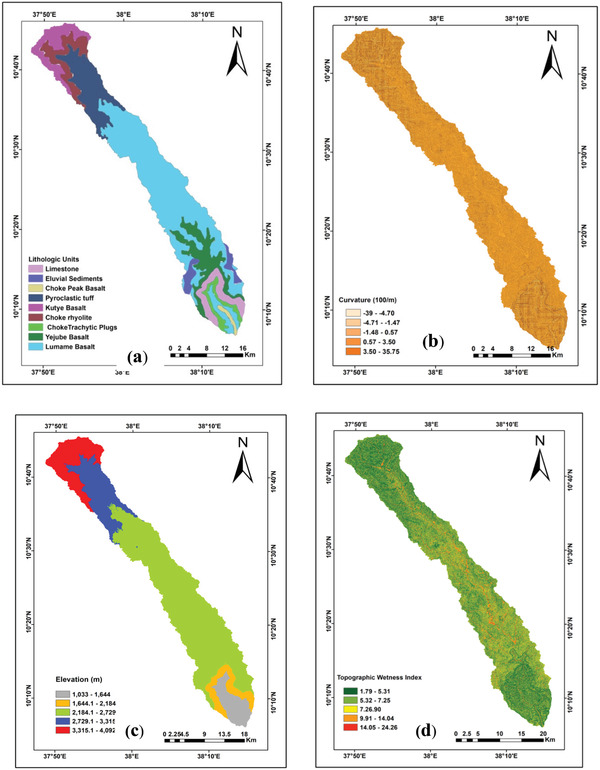
a) Lithological units of the study area, b) curvature of the study watershed, c) elevation of Muga watershed, and d) topographic wetness index (TWI).

Groundwater potential is highly determined by the occurrences of lithological features. The lithological features of the study area consist of basalts and trachyte, eluvial sediment, phyric basalt, limestone, and tuff (**Figure** **5**a and **Table** **1**). Basaltic rock is compact and hard in nature and insignificant in terms of permeability and porosity. A major part of the study area particularly the middle and lower course of the study watershed is covered by this kind of rock (Figure 5a). The weightage of lithology is assigned based on mineral, alteration, fractures, and weather conditions.

**Table 1:** Description of geological composition of the study area^[^ ^76^ ^]^

Geological formation	Description
Choke Shield Volcano/four major lava flows, one rhyolite lava flow, and trachyte plugs (22.4–23 Ma)	
Rob Gebaya Basalt	The phenocrysts proportion is variable: in terms of the phenocrysts proportion, it is plagioclase phyric basalt, olivine phyricbasalat, olivine plagioclase phyric basalt, olivine pyroxene plagioclase phyric basalt, pyroxene olivine phyric basalt respectively. The pyroclastic tuff trachyte sills interlayered with this unit.
AratMekerakr Basalt	Plagioclase phyric basalt and olivine plagioclase basalt form this unit. At places: the pyroclastic rock exposed at the base of this unit.
Kuy Basalt	Plagioclase phyric basalt and olivine plagioclase basalt form this unit. The trachyte sills have been injected into this unit.
Flood Basalts (four major lava flows (25.3–29.4 Ma))	
Arero Gidabo Basalt	Olivine phyricbasalat occasionally grades plagioclase olivine phyric basalt and olivine plagioclase phyric basalt and interlayered pyroclastic tuff. The top most of this unit is occupied by volcanic breccia (basaltic composition) or sandstone at places.
Yejube Basalt	Olivine plagioclase phyric basalt and plagioclase phyric basalt with occasional pyroxene plagioclase olivine phyric basalt pockets and interlayered with pyroclastic tuff. The top most of this unit at places overlaid by sandstone and ignimbrite or sandstone and pyroclastic tuff respectively.
Debre‐Markos Basalt	Olivine‐plagioclase‐phyric basalt, plagioclase olivine phyric basalt and olivine‐phyric basalt, one grades in to another. Occasionally the pyroclastic tuff interlayered with basalt. The top most of this unit overlaid by hill forming pyroclastic tuff on plateau.
Lumame Basalt	The phenocrysts proportion of this basalt is variable. The components of this basalt are olivine‐plagioclase‐phyric basalt, plagioclase‐phyric basalt, pyroxene plagioclase‐phyric basalt, pyroxene olivine ‐phyric basalt, pyroxene‐phyric basalt respectively. This unit locally interlayered with pyroclastic tuff. The top most of this unit overlaid by choke shield volcano and Islamo mountain Quaternary volcano.
Limestone	Fossiliferous limestone occasionally alternates with black shale impure (or black) shale limestone and very minor sandstone (Mesozoic era).
Lower sandstone (Adigrat sandstone)	Sandstone with lenses of conglomerate and siltstone and shale, overlaid by 40 m thick blue mudstone(Mesozoic era).
Lithologic units	
Eluvial sediments	

#### 3.1.8 Curvature

Intuitively, the curvature is the amount by which a curve deviates from being a straight line, or a surface deviates from being plane. Curvature is a topographical‐based factor, which shows the direction flow^[^ ^65^ ^]^ and clarifies at which rate the slope changes in the maximum slope direction.^[^ ^66^ ^]^ For the present study Curvature was generated from DEM as presented in Figure 5b.

#### 3.1.9 Elevation

Elevation is one of the topographic factors and considered as surface indicators to explore groundwater potential. The altitude of the study area was generated from STRM DEM and reclassified into five classes viz., 1033–1644, 1644–2184, 2184–2729, 2729–3315, 3315–4092 m (Figure 5c). Variation of altitude can alter climate conditions, this caused variation in rainfall, soil condition, vegetation, land uses, and vegetation type.^[^ ^67^ ^]^

#### 3.1.10 Topographic Wetness Index (TWI)

Topographic Wetness Index (TWI) plays a significant role in the hydrogeological system. TWI can explain the effect of topographic conditions on the size and location of saturated sources of surface runoff generation.^[^ ^45^ ^]^ Many researchers have been used TWI as a parameter to delineate the groundwater potential zones (e.g., refs. ^[^ ^46^, ^68^, ^69^, ^70^ ^]^).

The secondary topographic factor, TWI was calculated using the following equation^[^ ^68^ ^]^

(3) $$\begin{array}{c} \text{TWI}=\ln\left(\frac{\text{As}}{\tan\beta }\right) \end{array}$$

where As and tan β are the specific catchment area and the slope angle at the point, respectively. In the present study, TWI was classified into five classes (Figure 5d).

### 3.2 Generation of Weight for Groundwater Prospecting Parameters

#### 3.2.1 Analytical Hierarchical Process (AHP)

Analytical hierarchical process (AHP) model is one of a multicriteria decision‐making (MCDM) tool used to provide solutions for complicated decision‐making problems, and it was first introduced by.^[^ ^71^ ^]^ AHP is a widely accepted model used to assign a normalized weight for each thematic layer of groundwater prospecting factor. The final weight of each thematic layer was generated from the principal Eigenvalue of the generated matrix. The reliability of the output was determined by the calculated consistency index (CI) and consistency ratio (CR) values. The formula has been used

(4) $$\begin{array}{c} \text{CR}=\text{CI/RI} \end{array}$$

where CR indicates consistency ratio, RI indicates random consistency index whose values depend on the order of the matrix (**Table** **2**), and CI indicates consistency index which can also be calculated using the following formula

(5) $$\begin{array}{c} \text{CI}=\frac{\lambda \max-n}{n-1} \end{array}$$

where λ indicates the principal eigenvalue of the matrix and *n* is the number groundwater prospecting factors. The value of CR must be <0.1.

Experts have given comparison ratings based on Saaty's 1–9 scale (**Table** **3**) to determine the weight of each conditioning factor of prospecting parameters. The conditioning factors were compared against each other through a pairwise comparison matrix.

**Table 3:** Saaty's 1–9 scale of relative importance

Scale	Importance
1	Equal importance
2	Weak
3	Moderate importance
4	Moderate plus
5	Strong plus
6	Strong importance
7	Very strong importance
8	Very very strong importance
9	Extreme importance

**Table 2:** Saaty's ratio index for different values of *n*

N	1	2	3	4	5	6	7	8	9	10
RI	0	0	0.58	0.89	1.12	1.24	1.32	1.41	1.45	1.49

The inverse ranking method has been adopted to assign a normalized weight for each thematic layer. The potential of groundwater is represented by the rating of 1–5, where 1, 2, 3, 4, and 5 for very low, low, medium, high, and very high.^[^ ^72^, ^73^, ^74^ ^]^

#### 3.2.2 Groundwater Potential Index (GWPI)

The groundwater recharge potential map was generated by considering the comparative importance of various thematic layers and their corresponding classes. GWPI, a dimensionless quantitative approach was adopted to delineate groundwater potential zone.^[^ ^45^ ^]^

Considering all the themes of and features in an integrated layer, the groundwater potential index is calculated as

(6) $$\begin{array}{c} \begin{array}{l} \text{GWPI}={\text{Ge}}_{r}{\text{Ge}}_{w}+{\text{Rf}}_{r}{\text{Rf}}_{w}+{\text{Sl}}_{r}{\text{Sl}}_{w}+{\text{So}}_{r}{\text{So}}_{w}+{\text{Cu}}_{r}{\text{Cu}}_{w}\\ +\;{\text{TWI}}_{r}{\text{TWI}}_{w}+{\text{El}}_{r}{\text{El}}_{w}+{\text{DD}}_{r}{\text{DD}}_{w}+{\text{LD}}_{r}{\text{LD}}_{w}\\ +\;{\text{LULC}}_{r}{\text{LULU}}_{w} \end{array} \end{array}$$

where GWPI is groundwater potential index and the suffixes r and w represent the rank and weight of each layer.

## 4 Results

### 4.1 Weight Assigning and Normalization

In the present study, ten groundwater conditioning factors were identified and classified based on expert opinion and literatures. The experts’ knowledge was important to determine the rank of each conditioning factor. To determine the weight of each conditioning factor, questionnaires of comparisons ratings were prepared and filled by experienced experts. Using AHP model, the final geometric mean and feature normalized weight of each conditioning factor were presented in **Tables** **4** and **5**.

**Table 4:** Pairwise comparison matrix of 10 groundwater prospecting factors for the AHP model

Thematic layers	Ge	LD	DD	Rf	Sl	So	TWI	El	CU	LULC	Geometric mean
Ge	1.00	1.00	2.00	3.00	2.00	3.00	4.00	5.00	5.00	7.00	0.213028
LD	1.00	1.00	1.00	2.00	3.00	3.00	4.00	5.00	4.00	5.00	0.181301
DD	0.50	1.00	1.00	3.00	3.00	4.00	5.00	6.00	3.00	4.00	0.189672
Rf	0.33	0.50	0.33	1.00	2.00	3.00	5.00	5.00	3.00	4.00	0.124667
So	0.50	0.33	0.33	0.50	1.00	1.00	3.00	4.00	2.00	2.00	0.079353
Sl	0.33	0.33	0.25	0.33	1.00	1.00	3.00	3.00	1.00	3.00	0.067902
TWI	0.25	0.25	0.20	0.20	0.33	0.33	1.00	1.00	1.00	1.00	0.03345
El	0.20	0.20	0.17	0.20	0.25	0.33	1.00	1.00	2.00	3.00	0.039931
Cu	0.20	0.25	0.33	0.33	0.50	1.00	1.00	0.50	1.00	1.00	0.040033
LULC	0.14	0.20	0.25	0.25	0.50	0.33	1.00	0.33	1.00	1.00	0.030661
Sum (col)	4.4595	5.0667	5.8667	10.817	13.583	17	28	30.833	23	31	10.60112

Ge: geology; LD: lineament density; DD: drainage density; Sl: slope; So: soil; TWI: topographic wetness index; El: elevation; Cu: curvature; LULC: land use land cover.

**Table 5:** Weight assigning and normalization

Influencing factors	Feature	Assigned rank	Groundwater prospect	Feature normalized weight
Geology	Limestone	High	4	4/21 = 0.19
	Eluvial sediments	High	4	4/21 = 0.19
	Choke peak basalt	Low	1	1/21 = 0.047
	Pyroclastic tuff	Medium	3	3/21 = 0.14
	Kuye basalt	Low	2	0.09
	Choke rhyolite	Medium	3	0.14
	Choke trachytic plugs	Low	2	0.09
	Yejube basalt	Very low	1	0.047
	Lumame basalt	Very low	1	0.047
Total			21	
Lineament Density	−34.5 to −3.92	Low	2	0.11
	−3.92 to −1.16	Medium	3	0.16
	−1.16–1.03	High	4	0.22
	1.03–4.06	High	4	0.22
	4.06–35.75	Very high	5	0.27
Drainage Density	0–0.15	Very low	1	0.06
	0.15–0.47	Low	2	0.13
	0.47–0.67	Medium	3	0.2
	0.67–0.85	High	4	0.26
	0.85–0.95	Very high	5	0.33
Rainfall	1195 –1241 mm	Very low	1	0.06
	1242–1288	Low	2	0.13
	1289–1334	Medium	3	0.2
	1335–1380	High	4	0.26
	1381–1427	Very high	5	0.33
Soil	Chromic luvisols	High	4	0.23
	Chromic vertisols	Poor	2	0.11
	Eutric cambisols	Very Poor	1	0.05
	Eutric nitosols	Very High	5	0.29
	Leptosols	Medium	3	0.17
	Pellic vertisols	Poor	2	0.11
Slope	0–6.05	Very high	5	0.33
	6.06–13.25	High	4	0.26
	13.26– 22.47	Medium	3	0.2
	22.48–34.87	Low	2	0.13
	34.88– 73.49	Very low	1	0.06
TWI	1.79–5.31	Very high	5	0.33
	5.32–7.25	High	4	0.26
	7.26–9.90	Medium	3	0.2
	9.91–14.04	Low	2	0.13
	14.05–24.26	Very low	1	0.06
Elevation	1033–1.644 (m)	Very high	5	0.27
	1.644.1–2184	High	4	0.22
	2184.1–2729	Medium	3	0.16
	2729.1–3.315	Low	2	0.11
	3315.1–4092	High	4	0.22
Curvature	−39 to −4.70 (100 m^−1^)	Very low	1	0.076
	−4.69–1.47	Medium	3	0.23
	−1.46–0.57	High	4	0.307
	0.57–3.5	High	4	0.307
	3.5–35.75	Very low	1	0.076
LULC	Cultivated	Low	2	0.125
	Wetland	Very high	5	0.312
	Grassland	Medium	3	0.18
	Shrub land	Medium	3	0.18
	Bare land	Very low	1	0.06
	Forest	High	4	0.257

The consistency index (CI) and consistency ratio (CR) were cheeked while a pairwise comparison matrix of 10 groundwater prospecting factors was generated in AHP process. The principal Eigenvalue of the matrix is 10.601.

Hence, the consistency ratio (CR) = 0.0448 < 0.1. and the consistency index (CI) is 0.0668. For the model, where the AHP was used, the CR (consistency ratio) was performed. Based on the result which obtained in the analysis, the CR was found below 10%. The result was acceptable since the value of CR <0.1 is reasonable.

High lineament density and elevation indicate channeled runoff is concentrated in in the northern portion of Muga watershed. Based on lineament density classification medium, high and very high portion of is found in the northern and southern part of the study. The elevation map was divided in five classes and shown in Figure 5c; an elevation 1033–2184 m a.s.l. has less influence to groundwater occurrence. Based on pairwise comparison results, the rolling hill shape and flat terrain are performed as a higher weight and a mountainous shape was calculated as a lower weight. Low topographic relief as designated by low altitude, gentle slope and almost flat curvature indicate best infiltration conditions. The drainage density of the study area is reclassified in five classes: “very low” (0–0.15 km km^−2^), “low” (0.15–0.47 km km^−2^), and “medium” (0.47–0.670 km km^−2^), and “high” (0.67–0.85 km km^−2^), and “very high” (0.85–0.95 km km^−2^). Low and very low drainage density encircled large area of the study. The northern part receives rainfall of 1288–1427 mm whereas the vast portion receives below 1241 mm. The infiltration rate and the possibility of groundwater potential zones in the central and southern portion of the study area is directly influenced by the rainfall distribution of the northern area. The very high potential zone of groundwater is characterized by the lithology type such as limestone and eluvial sediments in Muga watershed.

## 5 Discussion

**Figure 6:**
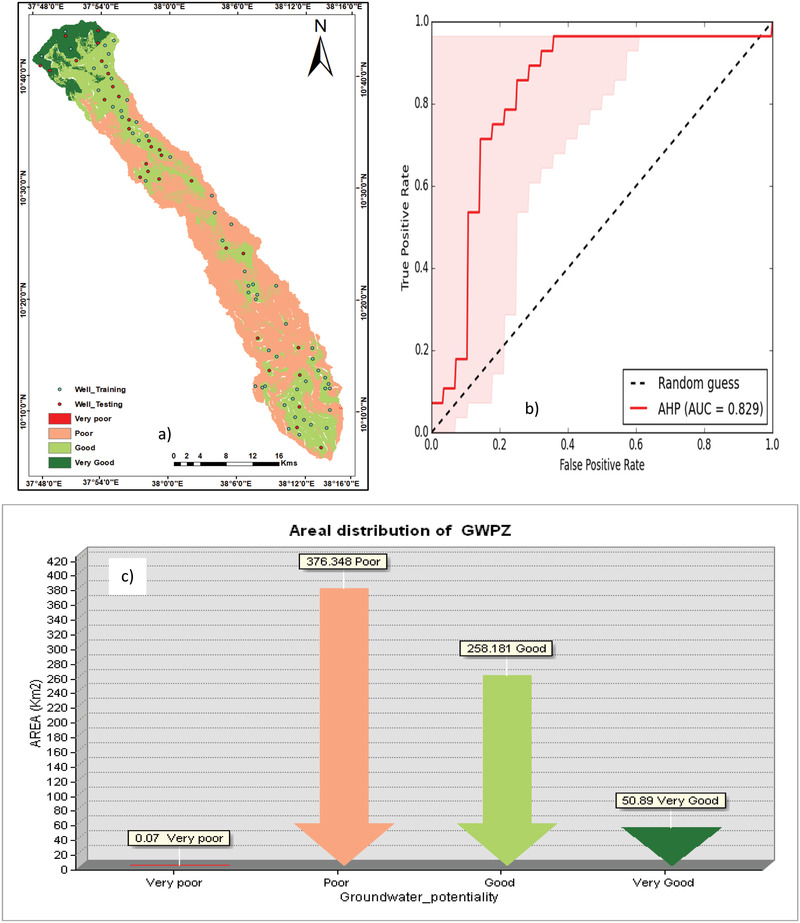
a) The overlaid map of training well, testing well and groundwater potential map, b) ROC curve for the groundwater potential map produced by AHP model, and c) areal distribution of groundwater potential zones

The final output of the model for the groundwater potential zones indicated that the potential recharge is strongly determined by such important parameters like lineament density, geology, elevation, slope etc. Based on the groundwater potential zone map (**Figure** **6**a) the northern part of the study area is characterized by high‐water storage on account of higher rainfall and lesser degree of slope. From the lithological point of view the Kutye basalt bears high amount of groundwater potential as compared to others in the northern part of study area. Forest coverage is dominantly found in northern part that can generate high potential groundwater. The result is similar with other study.^[^ ^23^ ^]^ The groundwater potential map generated by AHP model demonstrated acceptable result in predicting the groundwater recharge in Muga watershed, Ethiopia.

Validation is the most important process in the modeling of the resultant map of groundwater potential map developed by the thematic layers. In the present study, verification was done by considering the groundwater well locations from the datasets. Borewell and spring water data was generated and then overlaid groundwater recharge map of the study area. The Receiver operating characteristic curve (ROC) and area under curve (AUC) used to explore the prediction accuracy. The cumulative percentage of a potential map and the cumulative percentage of groundwater occurrence were used to generate the ROC curve. The qualitative and quantitative relationship between AUC value and the prediction accuracy can be grouped in five classes viz. poor (0.5–0.6), average (0.6–0.7), good (0.7–0.8), very good (0.8–0.9), and excellent (0.9–1).^[^ ^75^ ^]^ The groundwater potential map was validated with an 85 bore well. From the total of bore wells, 8 found over the very groundwater level. 74 boreholes fall under good water potential zone level. The rest of 3 boreholes were fallen over the moderate and poor groundwater potential zone. The ROC curve of the groundwater potential map indicates that very good value of AUC = 82.9 (Figure 6b) since it is between 0.8 and 0.9. Hence, the application of the AHP method for the present study showed reasonably very good accuracy spatial prediction of groundwater.

### 5.1 Distribution of Groundwater Potential and Its Implication for Groundwater Resources

The groundwater potential zones were estimated based on the weightage of individual features of thematic layers in the GIS environment. The GWPI values were adopted to classify whether an area of groundwater potential is very good, moderate, poor, and very poor. The total areal extent of the very high potential zone covers 52.7 km^2^ (7.4%). The good potential zone also covers 52.7 km^2^ (37.6%). The remaining parts comprise of poor and very poor zone which consists of about 387.9 km^2^ (55%) of the study watershed (Figure 6a,c).

The AHP map display groundwater distribution with high GWP potentials predominantly in the northern part of the study. Some portion of study area from north to south may have greater potential to exploit their groundwater resources. While the vast majority of the watershed is designated as poor and very poor, small areas of high and very high GWP exist across the northern and the southern tips in the study area. Western and eastern portion of Muga watershed is characterized by poor groundwater potential due to lesser rainfall and geological characteristics. The final result of groundwater potential map produced by AHP method is agreed with the bore wells yield data. Hence the movement and occurrence of groundwater in the study area is controlled by land use land cover, rainfall, elevation and drainage density as revealed from the result and checked directly from field observation. Based on the result, it is possible to develop sustainable groundwater management and irrigation practice since the place is an ideal for agriculture. Further investigations need to be carried out to assess groundwater salinity.

## 6 Conclusions

Assessment of groundwater potential is a vital step to use and manage resources effectively as well as efficiently. In the present study GIS, remote sensing and AHP technique proved to delineate GWPI like geology, rainfall, slope, soil, curvature, topography wetness index, elevation, drainage density, land use land cover, and lineament density in Muga watershed. The weight of thematic layers of groundwater prospect was depending on AHP process results.

The groundwater potential zones were obtained by overlaying all the thematic maps in terms of weighted overlay methods using the spatial analysis tool in ArcGIS 10.3, and it was found that the potential zones in terms of very high and good potential zones occupied an area of 52.7 and 265 km^2^, respectively. The remaining part comprises of poor and very poor zone that covers an area of 387.9 km^2^ of the study watershed. The area under curve (AUC) of ROC indicates the reasonable accuracy of a prediction system of the groundwater potential zone. In conclusion, the result of the groundwater potential map can serve as a base for planners in water resource management and land use planning. The application of geospatial technology with the integration of AHP techniques is a practical approach to groundwater prospecting and can be used in a similar environment.

## Conflict of Interest

The authors declare no conflict of interest.

## Author Contributions

All authors contributed to the study conception and design. Material design, data collection, and analysis were performed by T.M. and T.B. The first draft of the manuscript was written by T.M. and T.B. commented on previous versions of the manuscript. All authors read and approved the final manuscript.

## Data Availability

Research data are not shared.
